# β-adrenergic Receptor Stimulation Revealed a Novel Regulatory Pathway via Suppressing Histone Deacetylase 3 to Induce Uncoupling Protein 1 Expression in Mice Beige Adipocyte

**DOI:** 10.3390/ijms19082436

**Published:** 2018-08-17

**Authors:** Ana Yuliana, Huei-Fen Jheng, Satoko Kawarasaki, Wataru Nomura, Haruya Takahashi, Takeshi Ara, Teruo Kawada, Tsuyoshi Goto

**Affiliations:** 1 Laboratory of Molecular Function of Food, Division of Food Science and Biotechnology, Graduate School of Agriculture, Kyoto University, Gokasho, Uji, Kyoto 611-0011, Japan; anayulia@kais.kyoto-u.ac.jp (A.Y.); ydnas@kais.kyoto-u.ac.jp (H.-F.J.); kwrsk@kais.kyoto-u.ac.jp (S.K.); nom2@kais.kyoto-u.ac.jp (W.N.); haruya@kais.kyoto-u.ac.jp (H.T.); ara@kais.kyoto-u.ac.jp (T.A.); fat@kais.kyoto-u.ac.jp (T.K.); 2 Research Unit for Physiological Chemistry, the Center for the Promotion of Interdisciplinary Education and Research, Kyoto University, Kyoto 606-8501, Japan

**Keywords:** beige adipocyte, β-adrenergic receptor, histone deacetylase, histone acetylation, uncoupling protein 1

## Abstract

Browning of adipose tissue has been prescribed as a potential way to treat obesity, marked by the upregulation of uncoupling protein 1 (*Ucp1*). Several reports have suggested that histone deacetylase (HDAC) might regulate *Ucp1* by remodelling chromatin structure, although the mechanism remains unclear. Herein, we investigate the effect of β-adrenergic receptor (β-AR) activation on the chromatin state of beige adipocyte. β-AR-stimulated *Ucp1* expression via cold (in vivo) and isoproterenol (in vitro) resulted in acetylation of histone activation mark H3K27. H3K27 acetylation was also seen within *Ucp1* promoter upon isoproterenol addition, favouring open chromatin for *Ucp1* transcriptional activation. This result was found to be associated with the downregulation of class I HDAC mRNA, particularly *Hdac3* and *Hdac8*. Further investigation showed that although HDAC8 activity decreased, *Ucp1* expression was not altered when HDAC8 was activated or inhibited. In contrast, HDAC3 mRNA and protein levels were simultaneously downregulated upon isoproterenol addition, resulting in reduced recruitment of HDAC3 to the *Ucp1* enhancer region, causing an increased H3K27 acetylation for *Ucp1* upregulation. The importance of HDAC3 inhibition was confirmed through the enhanced *Ucp1* expression when the cells were treated with HDAC3 inhibitor. This study highlights the novel mechanism of HDAC3-regulated *Ucp1* expression during β-AR stimulation.

## 1. Introduction

Recently, browning of white adipose tissue (WAT) has garnered attention as a potent target for obesity. The term “browning” originated from the distinct characteristics of WAT and brown adipose tissue (BAT). WAT shows low oxidative capacity to support the storage of excess energy as triglyceride, while by contrast, BAT shows high oxidative capacity due to high mitochondrial content. Beige adipocyte develop within WAT in response to β-adrenergic receptor (β-AR) stimulation, such as cold conditions to activate a thermogenesis program to produce heat by increasing energy expenditure [1,2]. This phenomenon would be beneficial for obesity, as WAT has a relatively large mass, and therefore any change in cell physiology in this tissue may affect whole body metabolism. Once activated, beige adipocyte exhibit similar functional thermogenic characteristics as BAT, and thus browning is marked by upregulation of browning-fat specific genes, such as uncoupling protein 1 (*Ucp1*) [1,3,4]. The increased *Ucp1* expression in beige adipose tissue is especially evident in the inguinal region where basal levels of *Ucp1* are very low [5,6]. Many efforts have been made to pharmacologically stimulate the thermogenesis program through β-AR agonists, especially β3-AR. However, β3-AR agonists lack efficacy in human translational studies [7,8,9]. Hence, finding a new molecular target to induce browning is needed. 

Epigenetic modification has emerged as a new approach to treat diseases including cancer and obesity, by the remodelling of chromatin structure through mechanisms such as acetylation of lysine on histones. Histone is a protein that acts as a spool that packages DNA into nucleosomes and chromatin. Acetylated histone provides binding sites for transcription factors by neutralizing histone positive charges and loosening the interaction between histone and DNA, thus promoting gene expression [10]. Acetylation of histone 3 lysine 27 (H3K27ac) is known as a potent activation mark for browning-related genes, such as *Ucp1* [11,12,13,14]. H3K27ac distinguish active enhancers by allowing higher DNA access (open chromatin), which favours transcriptional activation [14,15,16]. Conversely, histone deacetylation condenses chromatin structure, suppressing gene expression [17,18]. Deacetylation of histone is mainly mediated by enzymes called histone deacetylase (HDAC) [18]. So far, there are 18 mammalian classical Zn^2+^-dependent HDAC that have been characterized and divided into four classes (class I–IV) based on similarity in structure to yeast HDAC [18]. Although structurally similar, the function of each HDAC homologue might differ depending on the complex HDAC formed. 

Interestingly, there is accumulating evidence that HDAC inhibitor (HDI) can stimulate browning of adipose tissue in BAT and WAT [11,12,19,20,21,22,23,24] which suggest the inhibition of HDAC might be closely related to browning. However, the detailed mechanism is still unclear and how β-AR stimulation might regulate HDAC activity is unknown. Indeed, in this study we show for the first time that β-AR stimulation also trigger similar HDAC inhibitory activity that benefited *Ucp1* expression in beige adipocyte.

## 2. Results

### 2.1. β-AR Stimulation-Induced Acetylation of Histone 3 Lysine 27 Favouring Open Chromatin Structure in the Ucp1 Promoter Region

**Figure 1:**
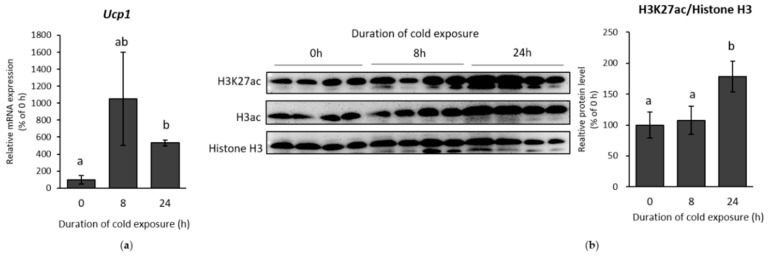
Histone acetylation state under cold stimulation in inguinal white adipose tissue (IWAT). (**a**) Uncoupling protein 1 (*Ucp1*) expression and (**b**) histone 3 lysine 27 acetylation (H3K27ac) level from the mice exposed to cold (10 °C) at different time points. Protein band were quantified by ImageJ. Data are presented as mean ± S.E.M. (error bars). *n* = 3–6 in each group. Different letters indicate significant difference (*p* < 0.05) according to one-way ANOVA followed by the Tukey-Kramer multiple comparison test. Same letters indicate non-significant difference.

**Figure 2:**
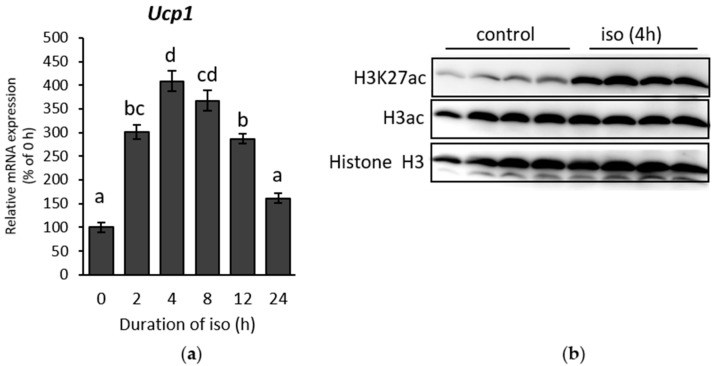

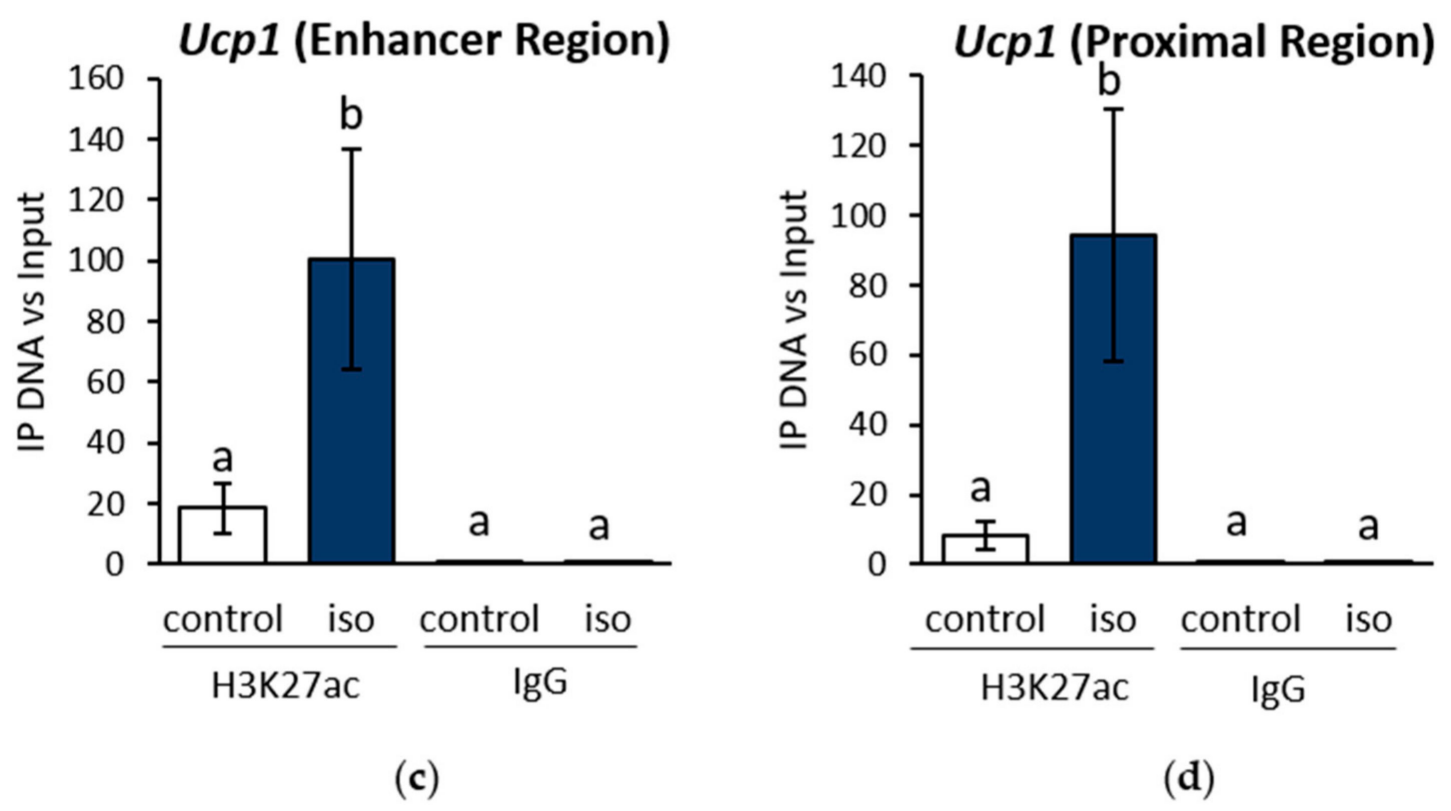
Histone acetylation state under β-adrenergic receptor (β-AR) stimulation in IWAT cell. (**a**) *Ucp1* expression after induction by 10 µM β-AR agonist isoproterenol (iso) over time, and H3K27ac level in (**b**) whole cells, (**c**) *Ucp1* enhancer region, and (**d**) *Ucp1* proximal region after induction by 10 µM β-AR agonist isoproterenol (iso) for 4 h. IgG act as a mock control. Data are presented as mean ± S.E.M. (error bars). *n* = 4–6 in each group. Different letters indicate significant difference (*p* < 0.05) according to one-way ANOVA followed by the Tukey-Kramer multiple comparison test. Same letters indicate non-significant difference.

β-AR were first stimulated by exposing mice to cold (10 °C) for 8 or 24 h. Inguinal white adipose tissue (IWAT) were then harvested. Browning in IWAT was successfully stimulated, indicated by the upregulation of *Ucp1* expression (Figure 1A). H3K27ac, a signature of histone activation, was also induced after 24 h cold exposure (Figure 1B). Furthermore, total histone H3 acetylation (H3ac), which includes several lysine sites, was also increased, suggesting a major transcriptional activation of genes during cold exposure. Similar results from the initial stage of cold exposure (0–24 h) in IWAT were also obtained in immortalized primary inguinal white adipose tissue cell (IWAT cell) treated with the β-AR agonist isoproterenol. The treatment showed an increment of *Ucp1* in time-dependent manner until 4 h (Figure 2A). On the other side, H3K27ac was also significantly increased when *Ucp1* was maximally expressed after 4 h isoproterenol addition (Figure 2B).

To further confirm the association between the increased histone acetylation in H3K27 and *Ucp1* upregulation during β-AR stimulation, a chromatin immunoprecipitation (ChIP) assay was performed to investigate if H3K27ac also increases within the *Ucp1* promoter region in IWAT cell. Two notable sites at the *Ucp1* promoter region (the enhancer and proximal regions) were selected to investigate histone acetylation in H3K27 and thus predict *Ucp1* chromatin state. The results showed that H3K27ac was considerably increased both in the *Ucp1* enhancer (Figure 2C) and proximal (Figure 2D) regions. In addition, peroxisome proliferator-activated receptor gamma coactivator 1 alpha (*Pgc1α*) mRNA upregulation upon β-AR stimulation, was also accompanied by an increase of H3K27ac in the *Pgc1α* of the cAMP response element (CRE) region, although the difference was not significant (Figure S1). This result may indicate that H3K27ac regulates not only *Ucp1* but other browning genes. Nevertheless, these results concluded an open chromatin structure for *Ucp1* transcriptional activation as marked by a significant increase of H3K27ac during β-AR stimulation in beige adipocyte.

### 2.2. β-AR-Stimulated Ucp1 Transcriptional Activation Is Associated with Inhibition of Class I But not Class II HDAC in IWAT Cell

**Figure 3:**
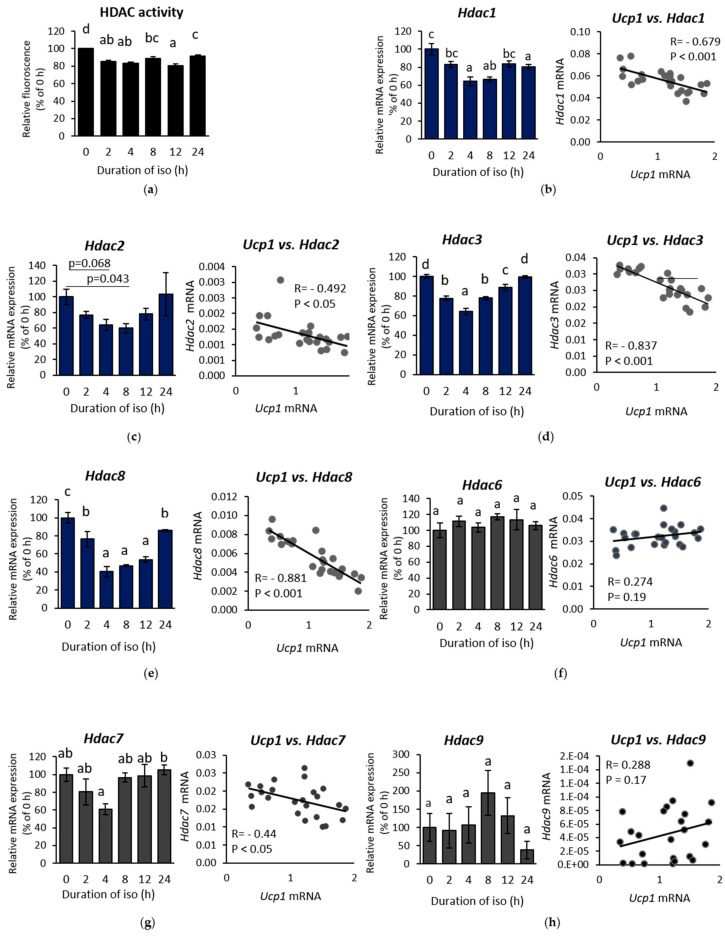
β-AR stimulation regulates histone deacetylase (HDAC) expression in IWAT cell. (**a**) HDAC activity, class I HDAC mRNA: (**b**) *Hdac1*, (**c**) *Hdac2*, (**d**) *Hdac3*, and (**e**) *Hdac8*; and class II HDAC mRNA: (**f**) *Hdac6* (**g**) *Hdac7*, and (**h**) *Hdac9* expression after induction by 10 µM iso in a time-course experiment. HDAC mRNA expression (left side) and its association with *Ucp1* (right side) based on Pearson’s correlation. Data are presented as mean ± S.E.M. (error bars). *n* = 4–8 in each group. Different letters indicate significant differences (*p* < 0.05) according to one-way ANOVA followed by the Tukey-Kramer multiple comparison test. Same letters indicate non-significant difference.

After confirming favourable chromatin state for *Ucp1* expression during β-AR stimulation, we investigated the role of HDAC, one of the main regulators of histone acetylation. HDAC activity was suppressed under isoproterenol induction (Figure 3A), displaying the opposite pattern to the level of *Ucp1* measured in the time-course experiment (Figure 2A). These data established a negative correlation between *Ucp1* and HDAC activity. To investigate the regulation behind HDAC inhibitory activity, we first examined the mRNA levels of class I and II HDACs, as HDIs, which previously reported to induce browning, mainly inhibit these two classes [17,25,26]. Almost all class I HDAC mRNAs, except *Hdac2*, were suppressed significantly upon isoproterenol treatment (Figure 3B–E, left side). In addition, Pearson’s correlation showed a strong negative correlation between class I HDAC mRNAs and *Ucp1* expression (Figure 3B–E, right side), which was consistent with the result from the measurement of HDAC activity (Figure 3A). On the contrary, class II HDAC mRNAs were barely altered and showed weaker correlation with *Ucp1* (Figure 3F–H). These results accentuate the importance of class I HDAC specificity, as confirmed by the remarkable increase of *Ucp1* expression after treatment with the class I HDAC inhibitor MS275 (Figure S2A), while also significantly decreasing HDAC activity (Figure S2B) in IWAT cell. Yet, among class I HDAC, *Hdac3* and *Hdac8* showed the highest correlation with *Ucp1* (Figure 3D,E right side). 

### 2.3. HDAC8 Might not Be Involved in Ucp1 Regulation in IWAT Cell

**Figure 4:**
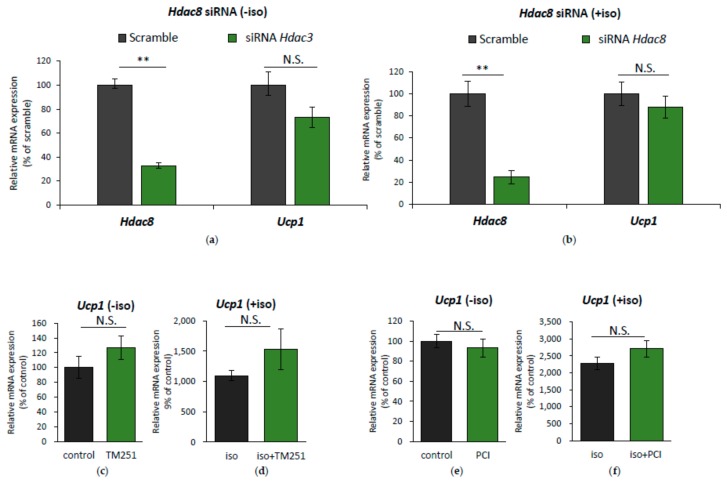
HDAC8 modification has no effect on *Ucp1* regulation in IWAT cell. *Hdac8* and *Ucp1* expression after transfection with *Hdac8* siRNA for 24 h without (**a**) or with (**b**) 10 µM iso induction for 2 h. *Ucp1* expression after treatment with 20 μM HDAC8 activator TM251 without (**c**) or with (**d**) 10 µM iso induction for 4 h. *Ucp1* expression after treatment with 5 μM HDAC8 inhibitor PCI34051 (PCI) for 24 h without (**e**) or with (**f**) 10 µM iso induction at the last 4 h. Data are presented as mean ± S.E.M. (error bars). *n* = 4–6 in each group. ** indicates significant difference (*p* < 0.01) according to unpaired-*t* test. N.S., not significant.

Of the two candidates of genes for *Ucp1* regulation, *Hdac3* and *Hdac8*, we first investigated the specific role of *Hdac8*. Initially, IWAT cell was transfected with *Hdac8* siRNA to mimic *Hdac8* downregulation, as shown by isoproterenol treatment, and *Ucp1* expression was measured. However, *Hdac8* siRNA interference failed to improve *Ucp1* expression, both on a basal level (Figure 4A) or under isoproterenol stimulation (Figure 4B). During isoproterenol induction, although *Hdac8* mRNA was downregulated (Figure 3E left side), HDAC8 protein level was barely changed (Figure S3), which might explain why *Ucp1* expression was unaffected by *Hdac8* siRNA. 

Previous reports have shown that HDAC8 activity is highly regulated through post-translational modification by phosphorylation, mediated by protein kinase A (PKA) [27,28], which is activated in β-AR stimulation [2]. Phosphorylation of HDAC8 resulted in a decrease of HDAC8 activity [27], which was shown upon isoproterenol treatment of IWAT cell (Figure S3), and this was further altered by PKA inhibition by H89. However, as HDAC8 activity was reduced, rescuing its activity by adding the HDAC8 coactivator TM251 barely altered *Ucp1* expression (Figure 4C,D). Additionally, IWAT cell was also treated with the selective HDAC8 inhibitor, PCI34051, under isoproterenol induction. Yet, we observed no significant change in *Ucp1* expression (Figure 4E,F). These data suggest that HDAC8 might not be involved in promoting *Ucp1* expression during β-AR stimulation. 

### 2.4. HDAC3 Inhibition Plays a Major Role in Ucp1 Transcriptional Activation During β-AR Stimulation in IWAT Cell

**Figure 5:**
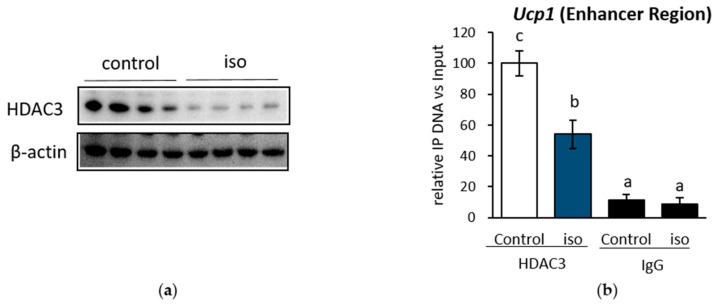
HDAC3 protein level was decreased under β-AR stimulation in IWAT cell. (**a**) HDAC3 protein level, and its recruitment level in (**b**) the *Ucp1* enhancer region after treatment with 10 μM iso for 4 h. Data are presented as mean ± S.E.M. (error bars). *n* = 4–6 in each group. β-actin act as loading control and IgG as a mock control. Different letters indicate significant differences (*p* < 0.05) according to one-way ANOVA followed by the Tukey-Kramer multiple comparison test. Same letters indicate non-significant difference.

When IWAT cell was treated with isoproterenol, *Hdac3* mRNA was significantly downregulated (Figure 3D, left side). Consistently, the level of HDAC3 protein was also reduced (Figure 5A). We investigated whether this decrease in HDAC3 protein level affected HDAC3 recruitment to the *Ucp1* enhancer region at the same site of induced histone activation mark H3K27ac, as shown in Figure 2C. The results showed that the HDAC3 recruitment level in the *Ucp1* enhancer region (Figure 5B) was inhibited significantly after isoproterenol addition. 

**Figure 6:**
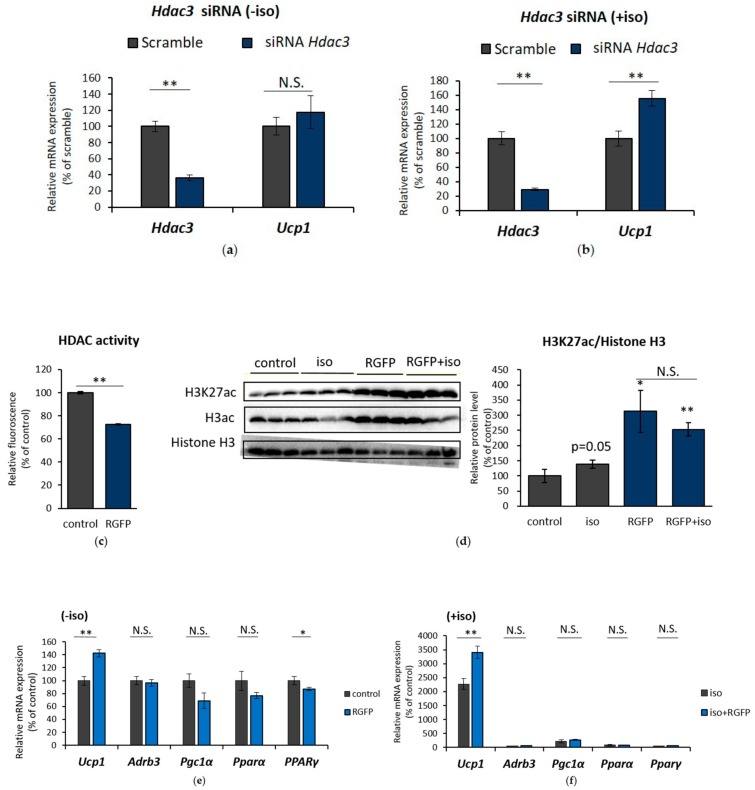
HDAC3 inhibition resulted in *Ucp1* upregulation in IWAT cell. *Hdac3* and *Ucp1* expression after transfection with *Hdac3* siRNA for 24 h without (**a**) or with (**b**) 10 µM iso induction for 2 h. (**c**) HDAC activity after treatment with HDAC3 inhibitor RGFP966 (RGFP) for 24 h. (**d**) H3K27ac level after induction by 10 µM iso for 4 h, 5 μM RGFP for 24 h, or both compounds. Protein band were quantified by ImageJ. Browning-related gene expression after treatment with 5 μM RGFP for 24 h without (**e**) or with (**f**) 10 µM iso induction in the last 4 h. Data are presented as mean ± S.E.M. (error bars). *n* = 3–6 in each group. *, ** indicate significant differences at *p* < 0.05 and *p* < 0.01, respectively, according to unpaired-*t* test. N.S., not significant.

Next, to specifically examine the role of *Hdac3* mRNA downregulation on *Ucp1* expression, *Hdac3* expression was interrupted by transfecting *Hdac3* siRNA into IWAT cell. Although not at the basal level (Figure 6A), *Hdac3* siRNA successfully increased *Ucp1* expression under isoproterenol stimulation (Figure 6B). Next, to further confirm the significance of HDAC3-specific inhibition of histone acetylation and *Ucp1* regulation, a selective inhibitor of HDAC3 (RGFP966) was used in IWAT cell. HDAC3 inhibitor treatment showed a significant decrease in HDAC activity (Figure 6C) and escalated H3K27 acetylation (Figure 6D). Unlike *Hdac3* siRNA, HDAC3 inhibitor alone was sufficient to induce *Ucp1* expression even at the basal level (Figure 6E), while also successfully enhancing *Ucp1* under isoproterenol induction (Figure 6F). Interestingly, neither adrenoceptor beta 3 (*Adrb3*), nor several influential coactivators of *Ucp1*, such as *Pgc1α*, *PPARα*, and *PPARγ* benefitted upon HDAC3 inhibition (Figure 6E,F), although class I HDAC inhibition by MS275 significantly upregulated *Adrb3* (Figure S2A). These results suggest that HDAC3 might be specifically and directly involved in *Ucp1* upregulation during β-AR stimulation.

## 3. Discussion

The favourable open chromatin structure for *Ucp1* transcriptional activation after β-AR stimulation has been previously reported in cultured brown adipocyte, demonstrated by a significant increase of H3K27ac in both the *Ucp1* and *Pgc1α* promoter regions [12]. However, which factors regulate behind this phenomenon is still unclear. The potential of HDAC, as one of the main regulators of histone acetylation, to regulate browning in adipose tissue has been suggested by several studies that tested HDAC inhibitory compounds in both BAT and WAT [11,20,24]. Moreover, many HDIs have shown to be beneficial in alleviating obesity and various diseases [20,21,22,23,29,30,31,32,33]. All of these reports suggest the possibility of β-AR activation to also possess HDAC inhibitory activity which could explain the escalated H3K27ac phenomenon under this stimulation. Indeed, we showed for the first time that HDAC was inhibited during β-AR stimulation in beige adipocyte (IWAT cell) and thus played an active role in *Ucp1* regulation, notably through HDAC3. HDAC3 was consistently suppressed at both the mRNA and protein levels, which resulted in the lower HDAC3 recruitment to the *Ucp1* enhancer region. The reduced HDAC3 recruitment thus directly cause an increased acetylation of H3K27 in the same site of *Ucp1* enhancer region and promote *Ucp1* transcriptional activation, as shown in this study. The *Ucp1* enhancer region (~2631–2343 bp upstream) is known as the peroxisome proliferator-activated receptor (PPAR) response element coactivated by PGC1α [34,35]. The suppression of HDAC3 in this region might promote PGC1α coactivator complex binding to the *Ucp1* enhancer and thus activate *Ucp1* transcription. 

Interestingly, *Hdac3* interference by siRNA did not result in *Ucp1* upregulation at the basal level, but improved *Ucp1* expression under isoproterenol stimulation, suggesting that *Hdac3*-regulated *Ucp1* transcriptional activation is dependent on β-AR stimulation. HDAC3 inhibitor treatment successfully enhanced *Ucp1* expression in both conditions. HDI decrease HDAC activity by disrupting the formation of the HDAC corepressor complex and hence it failed to be recruited to chromatin [36]. The same mechanism similarly happened to HDAC3 recruitment level to *Ucp1* promoter when stimulated with isoproterenol, as shown in this report. Thus, the reduction of HDAC3 recruitment level to the chromatin is important for *Ucp1* transcriptional activation, as its loss greatly affected histone acetylation state. HDAC3 might also specifically regulate *Ucp1* expression, because the treatment of HDAC3 inhibitor barely altered the mRNA expression of other browning markers such as *Adrb3*, *Pgc1α*, *Pparα*, and *Pparγ*. On the other hand, pan-HDAC inhibitor [20] or class I HDAC inhibitor MS275 (Figure S2) [24] was shown to upregulate not only *Ucp1*, but also *Adrb3* mRNA expression and other browning-related markers.

A previous report has shown that HDAC3-deficient mice underwent significant re-modelling of the WAT metabolic pathway to resemble BAT (browning) without β-AR stimulation [13]. This study thus highlights a negative regulation of HDAC3 to WAT browning. However, the reason why loss of HDAC3 was capable to induce browning remains unclear. Accordingly, our study clarifies the outcome of WAT browning in HDAC3 deficient mice might be because it has the same effect of HDAC3 suppression originated from β-AR stimulation to regulate *Ucp1* expression. Interestingly, the other study showed that the ablation of HDAC3 in mice during cold exposure (4 °C, 24 h) failed to activate the thermogenesis program in BAT [37]. In contrast, this study suggests a positive regulation of HDAC3 to browning. The different reports raise a question regarding the positive or negative role of HDAC3 in browning. To address the issue of the opposite role of HDAC3, our study showed that H3K27ac was also differentially regulated during initial and chronic stage of cold exposure. The level of H3K27ac increased at the initial stage of cold challenge (0–24 h, 10 °C) but decreased after long exposure (48–96 h, 10 °C), although *Ucp1* expression was stably increased (Figure S4). We thus suggest that the opposite HDAC3 regulation in browning might associate with a different regulation of H3K27ac during initial and chronic stage of cold exposure. Our in vitro study matched the initial stage of H3K27ac of cold stimulation in vivo. In the initial stage of cold exposure, the regulation of histone acetylation could be important for inducing *Ucp1* expression, as several studies [11,13], including this report, have suggested. Further research is needed to confirm the regulation of HDAC3 and H3K37ac in relation to *Ucp1* expression during chronic cold exposure.

Besides *Hdac3*, *Hdac1*, *Hdac7*, and *Hdac8* mRNA also showed a good association with *Ucp1* expression. However, HDAC1 and HDAC7 protein level were barely changed under isoproterenol treatment (Figure S5). In addition, HDAC1 specific inhibitor showed a negative effect on *Ucp1* expression. (Figure S6). Although HDAC1 deficiency has been reported to be involved in regulating BAT activation [12], it might be regulated differentially in beige adipocyte, as seen in this study. The role of HDAC1 in WAT browning, especially in beige adipocyte, should be confirmed in the future. Interestingly, isoproterenol treatment clearly mediated phosphorylation of HDAC8 through PKA, which has been established to decrease HDAC8 activity and resulted in hyperacetylation of histone H3 and H4 or non-histone protein [27]. It is also known that protein kinase A (PKA) is stimulated during β-AR stimulation, which resulted in the induction of *Ucp1* expression [2,3,5,38,39]. However, we found that HDAC8 might not be involved directly in *Ucp1* regulation. Additional research is needed to investigate the consequences of HDAC8 downregulation, particularly on non-histone substrates. Apart from 3600 possible acetylation sites (which served as HDAC substrate) that have been identified, 1750 site are non-histone protein [26,40,41,42,43]. Although we have demonstrated the HDAC inhibition in mediating acetylation of histone H3K27, the possibility of HDAC to also induce acetylation of non-histone substrate and their subsequent effect cannot be ruled out completely. 

Several pan-HDAC inhibitors have been approved for drug use and more HDAC inhibitors are under clinical trials, intended for cancer treatment [44]. Proportionally, HDAC inhibitor-based therapies have been recognized to be applicable to treat human disease. However, there is a concern over the side effects of pan-HDAC inhibitor [45], shifting the interest to more specific HDAC target. To address this issue, our study showed HDAC3 as a potential, more specific target for WAT browning. Many studies have also concluded HDAC3 as an emerging target for inflammation, insulin-resistance, and type 2 diabetes [31,46,47,48,49,50] which are closely related to obesity. Future study might be directed to the capability of the HDAC3 inhibitor to induce browning of WAT, as our study highly suggested, especially in humans. It has been known that browning of WAT has been targeted not only to treat obesity, but also related metabolic disorders including insulin resistance, inflammation and type-2 diabetes. Accordingly, our study added a solid background of the HDAC3 hidden potential in regulating *Ucp1* expression under β-AR stimulation. 

## 4. Materials and Methods

### 4.1. Materials

All chemicals were obtained from Nacalai Tesque (Nacalai Tesque, Kyoto, Japan), Wako (Wako Pure Chemicals, Osaka, Japan), Corning (Corning, Corning, NY, USA), Qiagen (Qiagen, Hilden, Germany), Invitrogen (Invitrogen, Carlsbad, CA, USA), and Sigma-Aldrich (Sigma-Aldrich, St. Louis, MO, USA). TM251 was purchased from Active Motif (Active Motif, Tokyo, Japan). PCI34051, and RGFP966 were acquired from Cayman (Cayman Chemical, Ann Arbor, MI, USA).

### 4.2. Animal Experiments

Mice were kept in a temperature-controlled room at 23 °C ± 1 °C with a 12 h light/dark cycle and free access to food (standard diet) and water. To stimulate β-AR activation, 14-week-old male C57BL/6N mice (SLC, Shizuoka, Japan) were exposed to cold (10 °C). Mice were sacrificed and inguinal white adipose tissue (IWAT) was harvested for mRNA and protein analysis. The mice were handled in accordance with procedures approved by the Animal Research committee of Kyoto University (Permission number: 29–62; 20 April 2012).

### 4.3. Cell Culture

Primary pre-adipocyte of IWAT was immortalized by transfecting pBabe-puro largeTcDNA retrovirus containing SV40 largeT antigen. Successfully transfected clone was screened based on puromycin resistance. Immortalized primary inguinal white adipose tissue cell (IWAT cell) was maintained in a humidified 5% CO_2_ atmosphere at 37 °C using basic medium (DMEM) supplemented with 10% fetal bovine serum and 1% penicillin/streptomycin. Maintenance medium consisted of basic medium supplemented with 0.25 μg/mL puromycin. Two days after confluence, cells were differentiated by stimulation with 0.5 mM 1-methyl-3-isobutylxanthine, 0.25 µM dexamethasone, 10 μg/mL insulin, 1 nM triiodo-l-thyronine (T3), 0.5 μM rosiglitazone (Rosi), and 125 µM indomethacin for 48 h. The media was then replaced by growth medium (basic medium containing 5 µg/mL insulin, 1 nM T3, and 0.5 µM Rosi) for another 48 h. After that, the media was changed every 2 days with basic medium supplemented with 5 µg/mL insulin and 1 nM T3. Generally, the differentiation process took 6–7 days. β-AR stimulation was induced by isoproterenol addition in serum free medium.

### 4.4. RNA Preparation and Quantification of Gene Expression

RNA was prepared and quantified as previously described [51,52]. Total RNA was extracted from cultured cells in 12-well plates or tissue according to the phenol-chloroform extraction method. RNA expression was quantified by real-time PCR using a LightCycler System (Roche Diagnostics, Mannheim, Germany) with SYBR Green fluorescence signal detection. All mRNA signals were normalized to a *36b4* internal control. The primer sequences are listed in Table 1.

### 4.5. Immunoblotting

Western blotting was performed as previously described [53]. Briefly, cells or tissue were lysed and protein was collected after centrifugation. Protein concentration was measured using the DC protein assay (Bio-Rad, Hercules, CA, USA). Denatured protein was then separated and transferred to a polyvinylidene difluoride transfer membrane. The membrane was then blocked, washed, and incubated with the corresponding primary antibody, followed by the appropriate secondary antibody. Anti-histone H3 (acetyl K27) (Abcam, Cambridge, UK), anti-acetyl-histone H3 (Millipore, Burlington, MA, USA), anti-histone H3 (Novus biological, Littleton, CO, USA), anti-HDAC3 (Sigma-Aldrich, St. Louis, MO, USA), and anti-β-actin (Cell Signaling Technology, Danvers, MA, USA) were used as primary antibodies. The secondary antibody staining was visualized using a chemiluminescent horseradish peroxidase (HRP) substrate (Millipore, Burlington, MA, USA).

### 4.6. HDAC Activity Assay

HDAC activity was measured using an HDAC Cell-Based Activity Assay Kit (Cayman Chemical, Ann Arbor, MI, USA) as described in the manufacturer’s instructions. Briefly, 1 × 10^4^ differentiated IWAT cell was plated in a 96-well black plate, clear bottom (Greiner Bio-One, Kremsmünster, Austria) as recommended by the protocol, and let to set for 6 h overnight before stimulation, and then analysed for HDAC activity.

### 4.7. Chromatin Immunoprecipitation (ChIP) Assay

The ChIP assay was performed according to company protocol (Upstate) with some modification. Cells were first fixed in 1% formaldehyde and then quenched by 125 mM glycine. Cells were collected and resuspended in 1% SDS lysis buffer, and then sonicated to shear DNA into 100–1000 bp fragments. The supernatant was collected and subjected to overnight immunoprecipitation with 4 µg H3K27ac antibody (Abcam, Cambridge, UK), 25 µg HDAC3 antibody (Abcam, Cambridge, UK), or rabbit IgG isotype control (Novus Biological, Littleton, CO, USA) as a mock control, together with Magna ChIP™ Protein A + G Magnetic Beads (Millipore, Burlington, MA, USA) at 4 °C in a rotatory shaker, followed by reverse cross-link and protease K digestion. Eluted DNA was then purified using a MinElute PCR Purification Kit (Qiagen, Hilden, Germany) and analysed by real-time PCR. Primer sequences are listed in Table 2.

### 4.8. Small Interfering RNA (siRNA) Transfection

The transfection of siRNA was performed in mature IWAT cell. After differentiation, cells were re-plated for 80% confluence in a 24-well plate. *Hdac3* and *Hdac8* siRNA (Table 3) transfection was performed according to the manufacturer’s instructions (Invitrogen, Carlsbad, CA, USA) using Lipofectamine 2000 transfection reagent (Invitrogen, Carlsbad, CA, USA). Cells were collected 24 h after transfection.

### 4.9. Statistical Analysis

All data were analyzed using Student’s *t*-test or one-way ANOVA followed by Tukey-Kramer test, when variances were heterogeneous. All data are presented as means ± SEM. Differences were considered significant at *p* < 0.05.

## Figures and Tables

**Table 1:** Primers used for RNA quantification.

Gene	Forward	Reverse
*Ucp1*	5′-CAAAGTCCGCCTTCAGATCC-3′	5′-AGCCGGCTGAGATCTTGTTT-3′
*Adrb3*	5′-GCACCTTAGGTCTCATTATGG-3′	5′-GCGAAAGTCCGGGCTGCGGCAGTA-3′
*Pgc1α*	5′-CCCTGCCATTGTTAAGACC-3′	5′-TGCTGCTGTTCCTGTTTTC-3′
*Pparα*	5′-TCGCGTACGGCAATGGCTTTT-3′	5′-CTTTCATCCCCAAGCGTAGGAGG-3′
*Pparγ*	5′-GGAGATCTCCAGTGATATCGACCA-3′	5′-ACGGCTTCTACGGATCGAAAACT-3′
*Hdac1*	5′-CCCATGAAGCCTCACCGAAT-3′	5′-CAAACACCGGACAGTCCTCA-3′
*Hdac2*	5′-CTGTCTCGCTGGTGTTTTGC-3′	5′-GTCATTTCTTCAGCAGTGGCT-3′
*Hdac3*	5′-ATGTGCCGCTTCCATTCTGA-3′	5′-TGGCATGATGTAGACCACCG-3′
*Hdac8*	5′-ACTTGACCGGGGTCATCCTA-3′	5′-AACCGCTTGCATCAACACAC-3′
*Hdac6*	5′-CAGCAGGATTTGCCCACCTA-3′	5′-TCTCCAGGACCTCCCAGAAG-3′
*Hdac7*	5′-TGGGGGATCCTGAGTACCTG-3′	5′-GTCCACCCTCTAAGGCCAAC-3′
*Hdac9*	5′-CCCACCACACATCACTGGAT-3′	5′-TCCATCCTTCCGCCTGAGTA-3′
*36B4*	5′-TCCTTCTTCCAGGCTTTGGG-3′	5′-GACACCCTCCAGAAAGCGAG-3′

**Table 2:** Primers Used in Chromatin Immunoprecipitation (ChIP) Assay.

Gene	Forward	Reverse
*Ucp1* enhancer	5′-CTCCTCTACAGCGTCACAGAGG-3′	5-AGTCTGAGGAAAGGGTTGA-3′
*Ucp1* proximal	5′-CCCACTAGCAGCTCTTTGGA-3′	5-CTGTGGAGCAGCTCAAAGGT-3′

**Table 3:** Small Interfering RNA (siRNA).

Gene	Sequence
*Hdac3*	CAGCAUGACAUGUGCCGCUUCCAUU
*Hdac8*	GACGGAAAUUUGACCGUAUUCUCUA

## Supplementary Materials

Supplementary materials can be found at http://www.mdpi.com/1422-0067/19/8/2436/s1.

## Abbreviations

β-AR	beta-adrenergic receptor
BAT	brown adipose tissue
ChIP	chromatin immunoprecipitation
CRE	cAMP response element
H3ac	histone 3 acetylation
H3K27	histone 3 lysine 27
HDAC	histone deacetylase
HDI	HDAC inhibitor
IWAT	inguinal white adipose tissue
PGC1α	peroxisome proliferator-activated receptor gamma coactivator 1 alpha
PKA	protein kinase A
PPAR	peroxisome proliferator-activated receptor
siRNA	small interfering RNA
UCP1	uncoupling protein 1
WAT	white adipose tissue
