# Supplementary material for: β-adrenergic Receptor Stimulation Revealed a Novel Regulatory Pathway via Suppressing Histone Deacetylase 3 to Induce Uncoupling Protein 1 Expression in Mice Beige Adipocyte
Source: Int J Mol Sci. 2018 Aug 17;19(8):2436. doi: 10.3390/ijms19082436 (PMC6121552; doi:10.3390/ijms19082436)
Supplement: Supplementary file 1 [file ijms-19-02436-s001.pdf]

Supplementary Material

Immunoprecipitation (IP) protocol-Cells were washed with PBS, and then lysed with IP buffer containing 25 mM Tris HCl pH 7.5, 150 mM NaCl, 1% NP-40, 0.1% SDS, 1 mM EDTA, and 1% protease & phosphatase inhibitor cocktail (Nacalai Tesque, USA). After centrifugation (13,200 rpm) at 4 °C for 10 min, cell lysates (2 mg proteins) were mixed with 1 μg HDAC8 antibody for 0.5 h at 4 °C and immune complexes were collected by incubation with protein G-sepharose beads (GE Healthcare, USA) for 1.5 h in the rotatory shaker (4 °C). The mixture was then centrifuged for 1 min at 700 x g (4 °C) and the precipitates were washed three times with IP buffer and then were treated with SDS-PAGE sample buffer. IP samples were denatured (5 min, 65 °C) and analyzed by western blotting using phospho-PKA substrate (RRCS*/T*) antibody to measure HDAC8 phosphorylation in Ser^{39} as the only one consensus of PKA recognition motif.

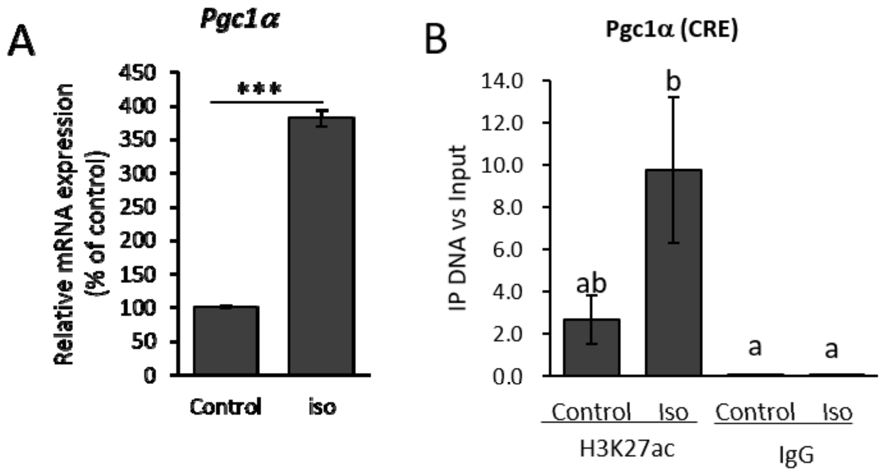

Figure S1. Histone acetylation state in *Pgc1α* promoter region under β-adrenergic receptor (β-AR) stimulation. (A) Peroxisome proliferator-activated receptor gamma coactivator 1-alpha (*Pgc1α*) expression and (B) H3K27ac level in *Pgc1α* cAMP response element (CRE) region in immortalized primary inguinal white adipose tissue cell (IWAT cell) after treated by 10 μM β-AR agonist isoproterenol (iso) for 4 h. *Pgc1α* signal were normalized to *36b4* internal control. Data are presented as mean ± S.E.M. (error bars). n = 4-6 in each group. *** indicates significant differences at $p < 0.001$ according to unpaired-t test. Different letters indicate significant difference ($p < 0.05$) according to one-way ANOVA followed by Tukey-Kramer multiple comparison test. Same letters indicate non-significant difference.

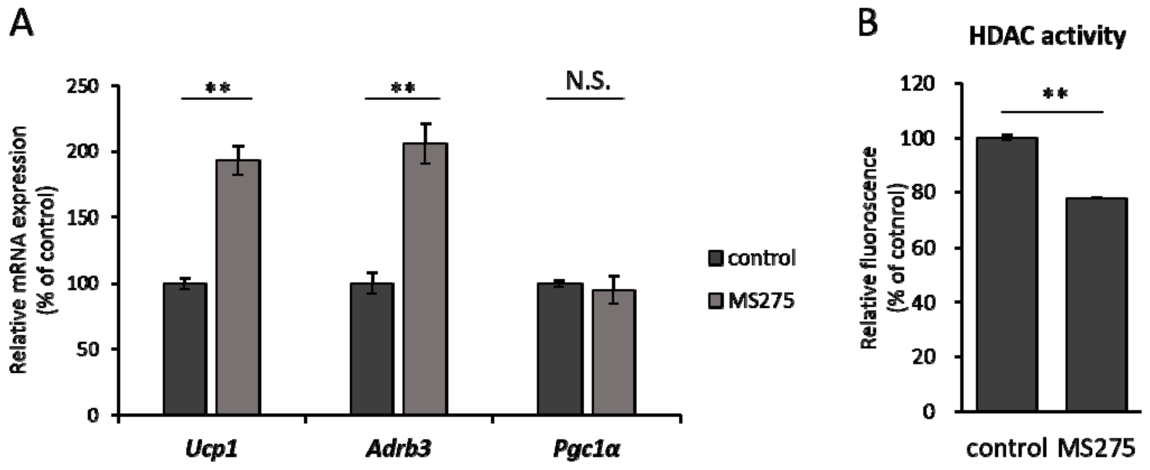

Figure S2. Class I HDAC inhibitor MS275 regulation on *Ucp1* expression in IWAT cell. (A) Browning-related gene expression and (B) HDAC activity after treated with 0.15 μM MS275 for 24 h. All mRNA signals were normalized to *36b4* internal control. Data are presented as mean ± S.E.M. (error bars). n = 4-8 in each group. ** indicates significant differences at $p < 0.01$ according to unpaired-t test. N.S., not significant.

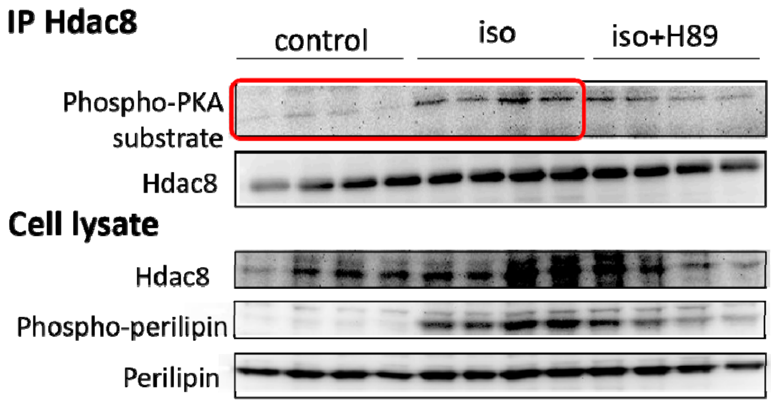

Figure S3. HDAC8 post-translational modification under isoproterenol stimulation in IWAT cell. Protein kinase A (PKA) phosphorylation of immunoprecipitated HDAC8 after induced by 10 µM iso or pre-incubated with 5 µM PKA inhibitor (H89) for 4.5 h (iso was added in the last 4 h of H89 incubation). *n* = 4 in each group.

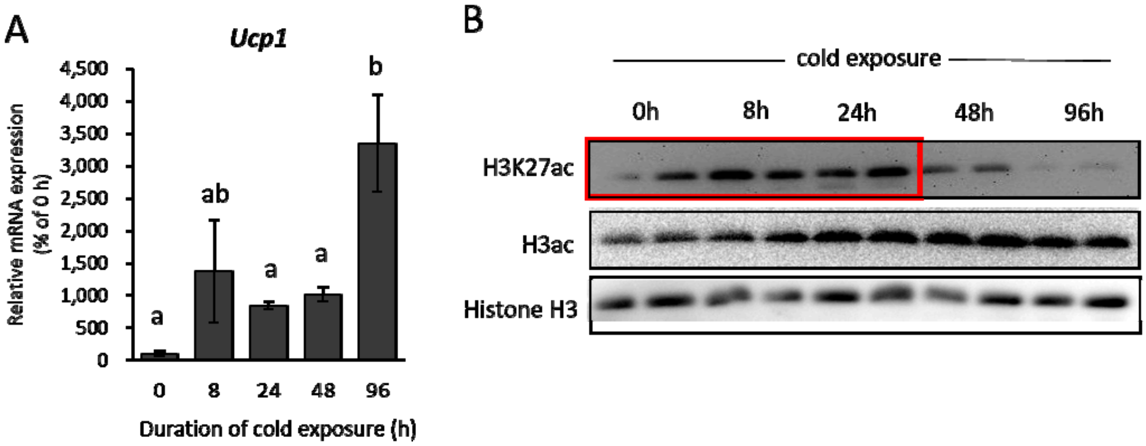

Figure S4. Histone acetylation state under β-AR stimulation in inguinal white adipose tissue (IWAT). (A) Uncoupling protein 1 (*Ucp1*) expression and (B) histone 3 lysine 27 acetylation (H3K27ac) level in IWAT. 14 weeks old male C57BL/6N were exposed to cold (10 °C) in different time point. *Ucp1* signals were normalized to *36b4* internal control. Data are presented as mean ± S.E.M. (error bars). *n* = 4 in each group. Different letters indicate significant difference ($p < 0.05$) according to one-way ANOVA followed by Tukey-Kramer multiple comparison test. Same letters indicate non-significant difference.

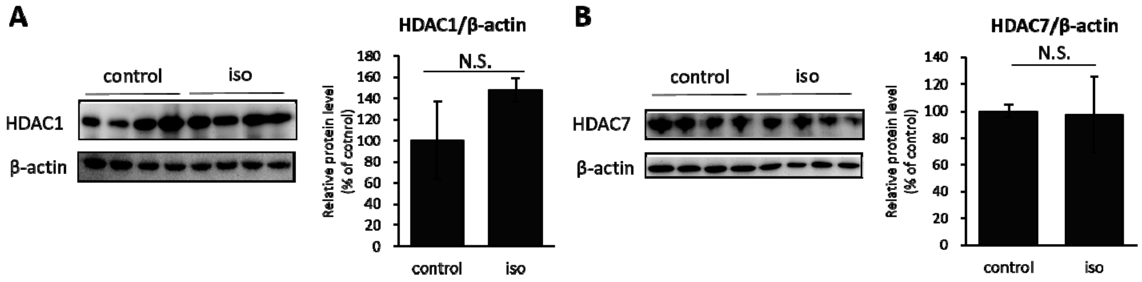

Figure S5. HDAC1 and HDAC7 protein level under β-AR stimulation in IWAT cell. *Left side*: (A) HDAC1 and (B) HDAC7 protein level after induced by 10 µM iso. *Right side*: protein quantification by ImageJ. Data are presented as mean ± S.E.M. (error bars). *n* = 4 in each group. N.S. indicate not significant according to unpaired-*t* test.

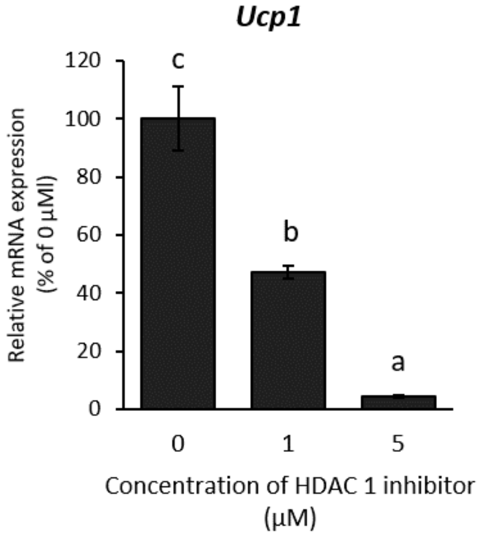

Figure S6. HDAC1 inhibition negatively regulate *Ucp1* mRNA expression in IWAT cell. *Ucp1* mRNA level after treated with HDAC1 inhibitor (CAY10398) in different concentration for 24 h. *Ucp1* signals were normalized to *36b4* internal control. Data are presented as mean ± S.E.M. (error bars). n = 4 in each group. Different letters indicate significant difference ($p < 0.05$) according to one-way ANOVA followed by Tukey-Kramer multiple comparison test. Same letters indicate non-significant difference.
